# Development, Production, and Postmarketing Surveillance of Hepatitis A Vaccines in China

**DOI:** 10.2188/jea.JE20130022

**Published:** 2014-05-05

**Authors:** Fuqiang Cui, Xiaofeng Liang, Fuzhen Wang, Hui Zheng, Yvan J Hutin, Weizhong Yang

**Affiliations:** 1 Chinese Center for Disease Control and Prevention, Beijing, China; 2 World Health Organization, China Office, Beijing, China (at the time this review was prepared and submitted)

**Keywords:** hepatitis A, vaccine, safety, efficacy

## Abstract

China has long experience using live attenuated and inactivated vaccines against hepatitis A virus (HAV) infection. We summarize this experience and provide recent data on adverse events after immunization (AEFIs) with hepatitis A vaccines in China. We reviewed the published literature (in Chinese and English) and the published Chinese regulatory documents on hepatitis A vaccine development, production, and postmarketing surveillance of AEFI. We described the safety, immunogenicity, and efficacy of hepatitis A vaccines and horizontal transmission of live HAV vaccine in China. In clinical trials, live HAV vaccine was associated with fever (0.4%–5% of vaccinees), rash (0%–1.1%), and elevated alanine aminotransferase (0.015%). Inactivated HAV vaccine was associated with fever (1%–8%), but no serious AEFIs were reported. Live HAV vaccine had seroconversion rates of 83% to 91%, while inactivated HAV vaccine had seroconversion rates of 95% to 100%. Community trials showed efficacy rates of 90% to 95% for live HAV and 95% to 100% for inactivated HAV vaccine. Postmarketing surveillance showed that HAV vaccination resulted in an AEFI incidence rate of 34 per million vaccinees, which accounted for 0.7% of adverse events reported to the China AEFI monitoring system. There was no difference in AEFI rates between live and inactivated HAV vaccines. Live and inactivated HAV vaccines manufactured in China were immunogenic, effective, and safe. Live HAV vaccine had substantial horizontal transmission due to vaccine virus shedding; thus, further monitoring of the safety of virus shedding is warranted.

## INTRODUCTION

In 1992, safe and effective vaccines for prevention of hepatitis A were licensed in a number of countries.^1^ Use of hepatitis A vaccine in several countries, including the United States, Israel, and Argentina, has led to a dramatic decrease in reported rates of hepatitis A.^2^ In 1988, the largest hepatitis A outbreak in the world (more than 310 000 cases) occurred in Shanghai, China. It heightened public concern^3^ and illustrated how accumulation of susceptible individuals after a decrease in transmission could result in large outbreaks of disease in the absence of vaccination-induced population immunity. In 1992, the world’s first live attenuated liquid hepatitis A vaccine was licensed in China.^4^ Beginning in 2000, a freeze-dried, more heat-stable, preparation progressively replaced the liquid form. In 2002, the first Chinese inactivated hepatitis A vaccine was licensed. The incidence of hepatitis A has decreased in all age groups, likely due to changing socioeconomic conditions and increasing hepatitis A vaccine use.^4^

In 2007, hepatitis A vaccine was fully integrated into the Chinese national immunization program and is provided free to all children older than 18 months. The Chinese national immunization program uses both the live attenuated vaccine (1 dose, subcutaneous route, about half of the market share) and the inactivated vaccine (2 doses, 6 months apart, about half of the market share). H2 strain vaccines have subsequently been licensed in India (2005), Guatemala (2006), the Philippines (2008), and Thailand (2010). The Chinese experience in developing and using the hepatitis A vaccine has not been widely shared, since most studies have been published in Chinese.

## LITERATURE REVIEW AND SURVEILLANCE

### Definitions

Immunogenicity, efficacy, and adverse events after immunization (AEFIs) were defined according to the recommendations of the World Health Organization and relevant position papers, consistent with common use in the published literature.^5^^–^^8^ Immunogenicity was defined as the ability of a hepatitis A vaccine to elicit an immune response of at least 20 mIU/mL. Efficacy was defined as a reduction in the incidence of a disease among people who have received a vaccine versus those who have not. An AEFI was defined as a post-immunization medical event that causes concern and is believed to be caused by the immunization.

### Literature review

We searched Chinese-language scientific journals and international journals using the key words: “hepatitis A”, “vaccine”, “immunogenicity”, and “safety”. To be included in the present review, the study must have (1) been an investigation of a vaccine produced in China, (2) included a control group, (3) included a denominator, nominator, or ratio that could be used to calculate rates, (4) described the duration time of follow up, rate of seroconversion, and geometric mean titer (GMT), and (5) been published between 1992 and 2010. We also reviewed official licensure documents and policy decisions regarding the use of hepatitis A vaccine in China.

### Surveillance data analysis

#### Data from the national AEFI surveillance system

In 2005, the China Centers for Disease Control (CDC) established the National AEFI Surveillance System on the basis of WHO guidelines.^8^ This system is a computer-based real-time system through which vaccination stations, clinics, manufacturers, parents, and the general public can report suspected AEFIs to county CDCs.

## DESCRIPTION OF VACCINES

### Overview of hepatitis A vaccines produced in China

In 1992, the first live attenuated hepatitis A vaccine was licensed. Chinese manufacturers currently produce live attenuated hepatitis A vaccine using the H2 strain (since 1992) and LA-1 strain (since 1994). In 2002, the first inactivated hepatitis A vaccine, similar in concept to those used internationally, was licensed in China. By 2010, there were 3 inactivated hepatitis A vaccines available in China: the LV-8 and TZ 84 strains and a Vero cell-based strain.^9^

#### H2 strain attenuated hepatitis A vaccines

*Development and licensure:* An attenuated live hepatitis A vaccine (H2 strain) was derived from a patient fecal specimen isolated in Hangzhou, China in the early 1980s. After isolation and passage in a culture of newborn monkey kidney cells, adaptation to growth in human lung diploid cells (KMB17), and serial passage at low temperature (32°C) in KMB17 cells, this strain became the master seed virus for the H2 strain vaccine.^10^ Two manufacturers used this H2 strain in a liquid preparation. In 2000, a freeze-dried preparation was licensed and replaced the liquid preparation. Both used the same H2 strain after cell culture, harvest, purification, and addition of a stabilizer (Table 1).

**Table 1.** Monovalent hepatitis A vaccines developed and produced in China

Type	Trade name	Attenuated HAV strain	Year licensed		Adjuvant	HAV antigen dose/injection		Manufacturers
	
			Liquid*	Freeze- dried		Pediatric (age, 1.5–15 years)	Adult (age, ≥16 years)	
Live-attenuated (initially in liquid form, now freeze-dried; the liquid form was removed from the market because the freeze-dried preparation is more stable)	Weisairuiji	H2	1992	2003	None	1.0 mL (6.50 lg CCID_50_)		Institute of Medical Biology of the Chinese Academy of Medical Sciences; Kunming

	Freeze-dried live attenuated hepatitis A vaccine	H2	1992	2003	None	0.5 mL (6.50 lg CCID_50_)	1 mL (6.50 lg CCID_50_)	Zhejiang Pukang Biotechnology Company Limited, Zhejiang Academy of Medical Sciences, Hangzhou

	Hepatitis A (live) vaccine, freeze dried	LA-1	1997	2000	None	1.0 mL (6.50 lg CCID_50_)		Changchun Institute of Biological Products & Changchun Changsheng Life Sciences Limited

	HAVAC	LA-1	1997	2000	None	1.0 mL (6.50 lg CCID_50_)		Changchun Institute of Biological Products

Inactivated	Healive	TZ84	2002	N/A	Aluminium hydroxide	250 units	500 units	Sinovac Biotech Co Ltd

	Weisairuian	Lv-8	2006	N/A	Aluminium hydroxide	320 ELISA units	640 ELISA units	Institute of Medical Biology of the Chinese Academy of Medical Sciences; Kunming

	Veraxim	YN5	2009	N/A	Aluminium hydroxide	800 ELISA units/0.5 mL	1600 ELISA units/1 mL	Shanghai Wison Bioengineering Inc

*not available at present.

*Production and quality assurance:* The freeze-dried, live attenuated hepatitis A vaccine is made with the H2 strain and KMB17 cell substrate and includes a protective additive that is patented in China. The vaccine comes in a 0.5-mL vial (for children) and a 1.0-mL vial (for adults) (Table 1). Administration is subcutaneous and shelf life is 18 months. During preparation, every master seed lot and working seed lot must pass a security test on monkeys, in accordance with China State Food and Drug Administration (SFDA) requirements. The KMB17 cell substrate is made of human embryonic lung diploid cells obtained from the Institute of Bio-Medical Sciences, Chinese Academy of Medical Sciences. In 2006, Good Manufacturing Practices (GMP) certificates were reissued for the 2 manufacturers. Between 2006 and 2009, the SFDA released 111 lots (about 9.85 million doses) of H2 strain vaccine.

#### LA-1 live attenuated hepatitis A vaccine

*Development and licensure:* In 1985, the virus precursor to the LA-1 strain was isolated from the stool of a 2-year-old boy with hepatitis in Heilongjiang Province, China.^11^ The Shanghai CDC and Changchun Institute of Biological Products collaborated to develop this into an attenuated strain.^11^ The China SFDA licensed a liquid form of the vaccine in 1997. In 2000, a freeze-dried preparation of the vaccine received a new drug license and marketing authorization and, in 2001, it was authorized for large-scale production, to replace the liquid form of the vaccine.

*Production and quality assurance:* Two manufacturers produce hepatitis A vaccine based on the LA-1 strain. The freeze dried live attenuated hepatitis A vaccine is made with the LA-1 strain and the 2BS cell substrate,^12^ which consists of human embryonic lung diploid cells (2BS) obtained from the national vaccine and serum institute. A patented protective additive includes trehalose, sodium glutamate, arginine, and dextran. The LA-1 vaccine comes in a 1.0-mL vial for subcutaneous administration and has a shelf life of 18 months. During preparation, every master seed lot and working seed lot must pass a security test on monkeys.^12^ In 2008, the GMP certificate was reissued. Between 2006 and 2009, SFDA released 164 lots (about 16 million doses of LA-1 strain vaccine).

#### Inactivated hepatitis vaccines

*Development and licensure:* In 2002, a domestically produced inactivated hepatitis A vaccine was licensed in China.^13^ This vaccine was prepared from the TZ84 strain derived from a fecal specimen of a patient who developed hepatitis A in Tangshan, Hebei Province in 1985. The virus used for vaccine production grew in human embryonic lung diploid fibroblast 2BS cells. Whole virus is extracted from tissue culture, purified, inactivated with formalin, and then adsorbed onto aluminium hydroxide. Sinovac Biotech Co Ltd developed and manufactures the TZ84 inactivated hepatitis A vaccine. In 2006, another domestic inactivated vaccine was licensed. The vaccine is prepared from the Lv-8 strain derived from the stool specimen of a 1988 patient from Shanghai. The strain was cultured in 25 passages, cultured in KMB17 cells for 12 days, harvested, purified, inactivated, and then adsorbed into aluminium hydroxide.^14^

*Production and quality assurance:* The steps involved in the manufacture of inactivated hepatitis A vaccine are similar to those for live attenuated vaccine. However, after extraction, there is an additional purification and inactivation step before the bulk vaccine is prepared.^15^ In 2008, the GMP certificate for the TZ84 vaccine was reissued. Between 2002 and 2009, the SFDA released 195 lots (more than 28 million doses of the TZ84 strain). Production of the Lv-8 strain is identical to that of the TZ84 strain, but the production period is shorter, which potentially increases production capacity^16^ (Table 1). In addition, the inactivated hepatitis A vaccine Veraxim (Vero cell) was licensed in China in 2009.

### Clinical trials

#### Safety of live attenuated hepatitis A vaccines

*Safety among recipients:* Initial clinical trials of live attenuated hepatitis A vaccines reported AEFIs including systemic fever (0.4% to 5%)^17^ and local rash (0% to 1.1%).^18^ Studies conducted between 1998 and 2003 described outcomes from a total of 589 940 recipients of live attenuated hepatitis A vaccines. Of the 3031 recipients of the H2 vaccine, 7 (0.23%) developed elevated aminotransferases and 2 (0.015%) developed other, minor symptoms.^19^

*Horizontal transmission:* Two studies^20^^,^^21^ examined the potential for horizontal transmission of live attenuated hepatitis A vaccine (H2) among a total of 42 vaccinated individuals and 75 contacts. One study investigated 38 recipients of the (H2 strain) vaccine. Virus was isolated from the stools of 34 recipients. Among the 75 contacts, 53 (71%) developed infection with vaccine virus isolated from the stools. Among the 42 recipients, none had an alanine aminotransferase level greater than 40 U/L; however, the overall mean alanine aminotransferase level increased from 12.84 U/L before vaccination to 16.49 U/L after vaccinated (*P* < 0.05).^20^ Two HAV-positive stools were isolated from recipients and 2 contacts and were injected into 8 monkeys. Six monkeys subsequently tested positive for vaccine virus in stool specimens; none developed abnormalities. Another study investigated 4 recipients of the H2 strain vaccine, and vaccine virus was isolated from 3 stools.^21^

### Safety of inactivated hepatitis A vaccines

*Safety among recipients:* Initial clinical trials of inactivated hepatitis A vaccines reported AEFIs, including systemic fever (1% to 8%). A review of the literature found no evidence of local rash or injection site induration.^22^ The types and rates of local and systemic reactions were similar after primary and booster doses, and no serious adverse reactions were noted.^23^ The domestic inactivated hepatitis A vaccine was also safe among recipients with liver disease.^24^ Passive surveillance data suggest that the incidences of AEFIs were similar to those of the live attenuated vaccine in China.^25^

### Immunogenicity

*Live attenuated vaccine:* With respect to the H2 vaccine, the median seroconversion rate for single doses greater than or equal to 6.5 log cell culture infectious doses (CCID) was 94% (range, 74%–100%) after 2 months and 91% (56%–100%) after 6 months. The GMT of anti-HAV antibody of initially susceptible subjects was 41 to 1607 mIU after 2 months, 75 to 119 mIU at 3 months, and 42 to 1945 mIU at 6 months. Overall, seroconversion rates were highest (median, 88%; range, 56%–100%) at 3 to 6 months after vaccination and were lower (median, 74%; range, 10%–100%) at 12 months. One study reported that antibodies persisted among 72% to 88% of individuals 15 years after 1 dose.^26^^,^^27^ Administration of a second dose of vaccine after 6 months led to seroconversion rates of 100% and higher GMTs.^28^ After a booster dose of H2 live attenuated vaccines, 100% of vaccine recipients seroconverted.^29^

For the LA-1 vaccine, the median seroconversion rate for doses greater than or equal to 6.5 log CCID was 78% (range, 74%–87%) at 3 months and 83% (83%–83%) at 6 months. The GMT of anti-HAV antibody among initially susceptible individuals was 90 to 122 mIU at 3 months and 80 mIU at 6 months, as reported in 3 studies.^30^^–^^32^ One study reported that antibodies persisted among 99% of individuals (71%–100%) at 3 years.^33^ In addition, an Indian study reported that freeze-dried live attenuated hepatitis A vaccine had good immunogenicity at 6 weeks (95.1% seroconversion) and 6 months (97.9% seroconversion)^34^ (Table 2).

**Table 2.** Studies of immunogenicity of live attenuated hepatitis A vaccines produced in China, 1997–2009

Study	Vaccine used	Subjects (No.)	Duration of follow up after first dose (months)	GMT (mIU)	Seroconversion, %	Dosage CCID_50_/mL	Number of doses received (schedule in months)	Age group (years)
Zhang Y, 1997^43^	H_2_	45	2.5	266	98	1.8 mL 10^6.83^	1	5–7
		52	2.5	102	90	0.9 mL 10^6.52^	1	
		39	2.5	81	80	0.4 mL 10^6.17^	1	
		59	2.5	188	92	0.9 mL 10^6.52^	1	18–22

Xu Z, 1998^30^	H_2_	102	3	87	92	10^7.0^	1	1–9
		101	6	91	87		1	

	LA-1	118	3	119	74	10^6.75^	1	
		95	6	81	83		1	

Xu Z, 1999^31^	H_2_	102	3	87	92	10^7.0^	1	1–9
		101	6	123	74		1	

	LA-1	119	3	91	87	10^6.75^	1	
		95	6	81	83		1	

Gong J, 1999^32^	LA-1	119	3	123	74	10^6.75^	1	1.5–10
		95	6	801	83		1	
		60	12	84	82		1	

Wang X, 2001^44^	H_2_	47	3	75	92	10^6.52^	1	6–8
		45	12	26	42		1	
		46	24	16	22		1	
		41	36	14	17		1	
		42	48	12	10		1	

Zhang X, 2002^45^	H_2_	69	0.5	49	80	10^6.5^	1	4–6
		66	2	55	83		1	
		68	6	80	92		1	
		68	9	65	88		1	
		38	12	83	92		1	
		57	24	134	95		1	

		42	0.5	31	60	10^6.5^	1	13–18
		42	2	41	74		1	
		39	6	42	85		1	
		39	9	489	87		1	
		37	24	33	78		1	

Zhuang F, 2003^17^	H_2_	94	2	1607	100	10^6.77^	1	7–12
		91	13	495	98		1	
		95	24	448	99		1	

	H_2_	114	2	1530	99	10^6.50^	1	3–6
		111	13	443	100		1	

Huang G, 2003^46^	H_2_	100	1	521	75	10^6.50^	1	6–12
		94	2	1607	100		1	

Xu G, 2003^18^	H_2_	68	2	228	91	10^6.5^	1	9–10

Wang X, 2004^28^	H_2_	26	1	19	31	10^5.5^	1	3–11
		26	6	47	58		1	

		26	1	1306	100	10^5.5^ and 10^6.83^	1	
		26	6	448	100		1	
		26	18	218	92		2 (0,6)

Zhuang F, 2005^47^	H_2_	220	2	287	99	10^6.5^	1	1–15
		219	12	226	94		1	
		176	72	173	83		1	
		155	120	145	80		1	

Faridi M, 2008^34^	H_2_	316	1.5	78	68	10^6.5^	1	1–5
		279	6	144	99		1	

Xu G, 2008^48^	H_2_	119	2	132	94	10^6.5^	1	2
		86	42	179	93		1	

Liu H, 2009^49^	H_2_	40	1	55	48	10^5.5^ and 10^6.83^	1	3–13
		37	6	83	62		1	
		37	7	1316	100		2 (0,6)	
		40	12	451	100		2 (0,6)	
		38	13	1945	100		3 (0,6,12)	
		33	24	899	100		3 (0,6,12)	
		40	84	337	100		3 (0,6,12)	

		43	1	47	33	10^5.5^ and 10^6.83^	1	
		43	6	72	56		1	
		44	7	1586	100		2 (0,6)	
		45	12	477	100		2 (0,6)	
		41	24	256	100		2 (0,6)	
		43	84	85	100		2 (0,6)	

CCID: cell culture infective dose 50%.

*Inactivated vaccine:* Studies of the immunogenicity of TZ84 vaccine at recommended doses for children and adults indicated that the median seroconversion rate was 87% (range, 62%–100%) at 1 month after a first dose and that GMT was 28 to 3630 mIU/mL. One month after administration of a booster dose, the seroconversion rate was 100% in 5 studies (GMT range, 264 to 21 696 mIU/mL) (Table 3). A double-blind randomized controlled trial compared TZ84 and a Glaxo Smith Kline (GSK) inactivated hepatitis A vaccine in healthy volunteers aged 1 to 8 years. Four hundred people were assigned to 4 groups. Each participant received 2 doses (at 0 and 6 months) of 1 of the 3 lots of TZ84 vaccine or the GSK vaccine. The seroconversion rate was 100% in all 4 groups at the end of the schedule. GMT ranged from 3237 to 3814 mIU/mL, and there were no significant differences between groups.^9^^,^^13^

**Table 3.** Studies of immunogenicity of inactivated hepatitis A vaccines produced in China (TZ84)

Study	Subjects (No.)	Duration of follow up after first dose (months)	Mean GMT	Seroconversion, %	Dosage	Number of doses received (months)	Age group (year)
Ren A, 2001^50^	16	1	139	94	1000 U	1	18–21
	16	3	138	100	1000 U	1	
	16	7	1067	100	1000 U	2 (0,6)	

Ren Y, 2002^51^	63	1	182	86	500 U	1	1–4
		3	243	89	500 U	1	
		6	280	84	500 U	1	

	37	7	4683	100	500 U	2 (0,6)	
	26	7	2718	100	500 U	2 (0,6)	

Liu C, 2002^52^	32	1	174	94	1000 U	1	5–15
	75	5	5160	100	1000 U	2 (0,3)	
	55	7	7540	100	1000 U	2 (0,6)	
	32	1	146	91	500 U	1	
	57	5	3269	100	500 U	2 (0,3)	
	50	5	4535	100	500 U	2 (0,3)	

Ren Y, 2002^53^	32	1	342	63	125 U	1	4–10
	33	1	382	94	250 U	1	
	33	1	391	100	250 U	1	
	32	6	531	100	125 U	2 (0,1)	
	32	7	3170	100	125 U	3 (0,1,6)	
	33	7	5963	100	250 U	2 (0,6)	

Ren A, 2002^23^	32	1	175	88	1000 U	1	18–20
	31	6	264	97	1000 U	1	
	32	7	2747	100	1000 U	2 (0,6)	
	40	1	159	70	500 U	1	
	26	6	259	65	500 U	1	
	40	7	1657	100	500 U	2 (0,6)	

Ren Y, 2003^54^	39	1	371	97	500 U	1	5–15
	49	1	320	100	500 U	1	
	70	1	321	99	500 U	1	
	33	1	355	97	250 U	1	
	38	1	195	95	250 U	1	
	67	1	251	96	250 U	1	
	31	5	6611	100	250 U	2 (0,3)	
	36	5	7154	100	250 U	2 (0,3)	
	39	3	2800	100	250 U	2 (0,1)	
	34	5	3265	100	250 U	2 (0,3)	
	33	7	5963	100	250 U	2 (0,6)	
	33	3	1973	100	250 U	2 (0,1)	

Ren Y, 2003^54^	34	3	417	100	250 U	1	5–10
	33	6	391	100	250 U	1	
	36	12	361	100	250 U	1	
	33	3	1973	100	250 U	2 (0,1)	
	34	5	3265	100	250 U	2 (0,3)	
	33	7	5963	100	250 U	2 (0,6)	
	36	7	14 893	100	250 U	2 (0,12)	

Ren Y, 2003^54^	36	13	195	94	250 U	1	4–10
	36	12	360	100	250 U	1	
	49	1	370	100	500 U	1	
	49	12	456	100	500 U	1	
	36	13	14 893	100	250 U	2 (0,12)	
	49	13	21 696	100	500 U	2 (0,12)	

Li Y, 2003^55^	97	1	758.6	95	720 EIU	1	Children (average age, 9.7 years)
	105	6	2951	99	720 EIU	1	
	99	7	10 471	100	720 EIU	2 (0,6)	

	120	1	3631	97	1440 EIU	1	Adults (average age, 17.2 years)
	119	6	2455	100	1440 EIU	1	

Ren Y, 2003^54^	52	12	869	100	500 U	2 (0,3)	5–10
	58	24	782	100	500 U	2 (0,3)	
	58	36	379	100	500 U	2 (0,3)	
	55	12	981	100	500 U	2 (0,6)	
	55	24	667	100	500 U	2 (0,6)	
	55	36	462	100	500 U	2 (0,6)	

Yao J, 2004^56^	73	2	260	95	250 U	1	2–3

Jia X, 2004^57^	91	1	14 407	100	250 U	1	2–15

Jiang W, 2008^9^	122	1	28	75	250 U	1	1–3
	125	6	118	97	250 U	1	
	121	7	3106	100	250 U	2 (0,6)	

	167	1	29	75	250 U	1	4–8
	168	6	103	97	250 U	1	
	166	7	3686	100	250 U	2 (0,6)	

Veraxim inactivated hepatitis A vaccine (Vero cells) is the first inactivated hepatitis A vaccine in the world to be based on Vero cell culture. It was licensed by the SFDA in 2009. Phase III clinical trial results showed a seroconversion rate of 98% among children with a vaccination schedule of 0 and 6 months apart (with doses of 800 ELISA units/0.5 mL/dose), and a rate of 97% among adults (with doses of 1600 ELISA units/1 mL/dose). There was no significant difference in systemic or local reaction rates between children (10.60% and 2.28%, respectively) and a control group (10.71% and 2.86%, respectively). In addition, systemic (8.80%) and local reaction rates (2.67%) in adults did not differ from those in a control group. However, postmarketing surveillance data are not available.^35^

### Efficacy

#### Live attenuated vaccine

The protective efficacy against infection of the H2 and LA-1 vaccines exceeded 90% in community trials (Table 4).^36^ In a community-based cohort study, the vaccine had 95% protective efficacy against outbreaks in a population where vaccine was used. A meta-analysis of the efficacy of live attenuated hepatitis A vaccine (H2 and LA-1), which included 13 randomized trials, showed that the protective efficacy was 96% (95% CI, 93%–98%), which is equivalent to that of international inactivated vaccines.^37^

**Table 4.** Cohort studies of efficacy of hepatitis A vaccines produced in China

Study	Vaccine	Recruitment: No. of subject in each group		Follow up: No. of hepatitis A cases in each group		Vaccine efficacy, %	Follow-up (years)
	
		Vaccinated	Unvaccinated	Vaccinated	Unvaccinated		
Mao J, 1997^27^	H2	18 102 person-years	242 168 person-years	0	495	100	4.0
Xu Z, 1998^30^	LAV-1	260 117	235 235	0	37	100	0.5
Li Y, 2000^58^	LA-1	208 328	192 448	5	66	93	1.0
		84 412	79 254	0	22	100	1.0
Zhang Y, 2001^59^	H2	10 459	13 005	1	20	94	3.5
Zhuang F, 2001^26^	H2	745	475	0	11	100	10.0
Ma J, 2002^60^	H2	1804	1653	1	14	94	3.5
Gong J, 2003^33^	LA-1	3771	3545	2	71	97	3.0
Luo D, 2004^61^	LA-1	15 779	60 517	1	38	90	5.0
Wang X, 2000^62^	H2	21	237	1	119	98	1.0

Randomized controlled studies during the period 1997 through 2003 (duration of follow up, 6 months to 10 years) found that the efficacy of live attenuated hepatitis A vaccines (H2 strain) produced in China was 95% to 100%.^42^^–^^53^ An LA-1 strain hepatitis A vaccine showed a protective efficacy of 95% to 100% after 2 years of follow up.^38^

#### Inactivated hepatitis A vaccine

A non-randomized controlled trial evaluated the protective efficacy of TZ84 inactivated hepatitis A vaccine during a hepatitis A outbreak. No hepatitis A cases were detected among the 3365 students who received the vaccine, while 4 cases of hepatitis A were reported in the control group of 2527 people (protective efficacy, 100%).^39^

### Postmarketing surveillance, including AEFI surveillance

#### Postmarketing surveillance

As of 2010, freeze-dried live attenuated hepatitis vaccine had been given to more than 80 million persons. Only 12 cases of mild reactions were reported directly to the manufacturers from 2007 through 2009, and no serious reactions were reported. In 2009, postmarketing surveillance data were reported for H2 vaccine given to 562 vaccinees in Jiangsu province: 35 (6%) cases of mild fever were reported, but no local reactions were noted.^40^ Postmarking surveillance data are not available for the LA-1 vaccine.

#### AEFI surveillance

From 2005 through 2009, 80 382 AEFIs associated with any vaccine were reported to the China CDC through the national AEFI surveillance system. Of these, the most frequently reported were fever, local erythema, local swelling, and local sclerosis, which accounted for 78% of all AEFIs reported. Other AEFIs were anaphylaxis (9%) and aseptic abscess (4%). No fatal AEFIs and no clusters of AEFIs were reported. Overall, during 2005–2009, AEFIs among recipients of live attenuated hepatitis A vaccine accounted for 0.7% of all AEFIs reported in China. Analysis of AEFI rates by doses distributed suggests that the rates did not differ between inactivated vaccine (34 AEFI/million doses) and live attenuated vaccine (36 AEFI/million doses).^25^^,^^41^

## COMMENT

Studies in China indicate that the dose of live attenuated hepatitis A vaccine should be at least 10^6.5^ CCID_50_/mL to obtain a satisfactory immune response.^28^^–^^42^ While immune responses to international inactivated vaccines lead to seroconversion in more than 95% of vaccinees at 2 weeks, Chinese live attenuated vaccines may require more time to generate antibodies.^37^ Field studies indicate that efficacy is 90% to 100%. With respect to inactivated vaccine, there was variation in the proportion of vaccinees who had seroconverted at 1 month. However, 100% of recipients seroconverted after administration of a 2-dose series.^42^

Initial Chinese trials of live attenuated and inactivated hepatitis A vaccines revealed no vaccine safety issues except for mild fever and local rash and indurations at the injection site. However, person-to-person transmission of live attenuated vaccine virus remains a safety concern because vaccine recipients excrete virus and transmit it to a high proportion of their contacts. Such human-to human transmission has the theoretical potential of vaccine virus mutation, including possible reversion to the wild type, as is seen with live attenuated poliovirus vaccine. Studies conducted so far have suggested that live hepatitis A vaccine virus has hereditary stability and that the vaccine strain is stable. No reversal to virulence has been documented. In addition, all infections with the attenuated virus have been asymptomatic and aminotransferases remained within normal ranges. However, there have been no studies of the practical consequence of horizontal transmission in the field, including outcomes of secondary infection and the consequences of circulation of attenuated virus.

We have several recommendations. First, we must further quantify the field effectiveness of Chinese hepatitis A vaccines. This will require precise coverage estimates, additional field investigations, and epidemiologic modeling. Investigation of post-exposure prophylaxis would also be of interest so that comparisons can be made with other hepatitis A vaccines. Second, the safety profile of the Chinese hepatitis A vaccine needs to be further documented. This will require analyses of AEFIs and calculation of AEFI rates by vaccine dose. Rigorous, high-quality postmarketing surveillance in selected communities, to measure and monitor safety and adverse reactions, will also be useful. Elements of particular interest are (1) identification of molecular markers of attenuation, (2) documentation of the genetic stability of these markers of attenuation after human passages, and (3) safety of orally ingested vaccine virus. Well-conducted large-scale clinical trials of efficacy and clinical safety will provide more robust documentation of the effectiveness and safety of Chinese hepatitis A vaccines. In view of the large volume of live hepatitis A vaccines used in China, and their potential usefulness outside China, carefully collected and validated data on safety and efficacy will be valuable. Such evidence will be a key component of a strategy to make the vaccines available for use internationally as part of global hepatitis A prevention efforts.^53^
